# Casein Kinase 2 Signaling in White Matter Stroke

**DOI:** 10.3389/fmolb.2022.908521

**Published:** 2022-07-13

**Authors:** Hung Nguyen, Wenbin Zhu, Selva Baltan

**Affiliations:** Anesthesiology and Peri-Operative Medicine (APOM), Oregon Health and Science University, Portland, OR, United States

**Keywords:** mitochondria, micro RNA, CX-4945, silmitasertib, akt, post-ischemic protection, ischemia

## Abstract

The growth of the aging population, together with improved stroke care, has resulted in an increase in stroke survivors and a rise in recurrent events. Axonal injury and white matter (WM) dysfunction are responsible for much of the disability observed after stroke. The mechanisms of WM injury are distinct compared to gray matter and change with age. Therefore, an ideal stroke therapeutic must restore neuronal and axonal function when applied before or after a stroke, and it must also protect across age groups. Casein kinase 2 (CK2), is expressed in the brain, including WM, and is regulated during the development and numerous disease conditions such as cancer and ischemia. CK2 activation in WM mediates ischemic injury by activating the Cdk5 and AKT/GSK3β signaling pathways. Consequently, CK2 inhibition using the small molecule inhibitor CX-4945 (Silmitasertib) correlates with preservation of oligodendrocytes, conservation of axon structure, and axonal mitochondria, leading to improved functional recovery. Remarkably, CK2 inhibition promotes WM function when applied after ischemic injury by specifically regulating the AKT/GSK3β pathways. The blockade of the active conformation of AKT confers post-ischemic protection to young and old WM by preserving mitochondria, implying AKT as a common therapeutic target across age groups. Using a NanoString nCounter miRNA expression profiling, comparative analyses of ischemic WM with or without CX-4945 treatment reveal that miRNAs are expressed at high levels in WM after ischemia, and CX-4945 differentially regulates some of these miRNAs. Therefore, we propose that miRNA regulation may be one of the protective actions of CX-4945 against WM ischemic injury. Silmitasertib is FDA approved and currently in use for cancer and Covid patients; therefore, it is plausible to repurpose CK2 inhibitors for stroke patients.

## Introduction

### The Role of Casein Kinase 2 in Brain Ischemia

Casein Kinase 2 (CK2), is an unconventional protein kinase (PK) that is composed of two catalytic α-subunits (α and α’) and two β-subunits (Pinna and Allende, 2009). CK2 is shown to phosphorylate numerous substrates, including other PKs, thus acting as a “master regulator” (Meggio and Pinna, 2003; Poole et al., 2005). Unlike other PKs, the catalytic activity of CK2 is not regulated by second messengers or phosphorylation (Poole et al., 2005; Vilk et al., 2008) and it is constitutively active. In addition, CK2 is recently reported to be activated *via* a polymerization/depolymerization mechanisms (Kristensen et al., 2004; Lolli et al., 2012; Franchin et al., 2018) together with reactive oxygen species (ROS) (Leslie and Downes, 2002; Cosentino-Gomes et al., 2012; Srinivasan et al., 2013). CK2 regulates many cellular functions and its activity is vital for brain development (Lou et al., 2008) and cellular homeostasis (Blanquet, 2000; Brunet et al., 2015). For instance, direct interaction of CK2β with the transcription factor Olig2 is required for oligodendrocyte progenitor cell development and lineage (Huillard et al., 2010). On the other hand, upregulation of CK2 signaling is linked to many diseases, such as cancers (Sarno et al., 2002), cardiac hypertrophy (Hauck et al., 2008; Eom et al., 2011), and ischemic injury (Hu et al., 1993; Hu and Wieloch, 1993; Ka et al., 2015).

CK2 is constitutively expressed in the central nervous system (CNS) including glial cells such as adult oligodendrocytes (Huillard et al., 2010) and has a complex role in cellular injury (Ka et al., 2015). A previous report has shown that CK2 is a neuroprotectant that acts by directly modulating NADPH oxidase activity during cerebral ischemia (Kim et al., 2009). On the other hand, a brief and moderate AMPA receptor activation in rat oligodendrocyte cell cultures triggers CK2 activity to mediate excitotoxic injury (Canedo-Antelo et al., 2018). Accordingly, CK2 inhibition alleviates AMPA-mediated excitotoxic oligodendrocyte death by blocking AMPA receptor activation (Canedo-Antelo et al., 2019). Although these findings propose an intriguing role for CK2 signaling in brain ischemic injury mechanisms, the role of CK2 has remained unexplored in white matter (WM) function and ischemic injury mechanisms until recently (Baltan et al., 2018; Bastian et al., 2019b). WM is composed of astrocytes, oligodendrocytes, microglia, axons, and myelin that wraps them (Fields, 2008) therefore an ideal stroke therapeutic must be directed towards neurons, axons, and glial cells across all age groups. Currently, recombinant tissue plasminogen activator or endovascular thrombectomy are effective treatments for reperfusion after ischemic stroke. However, reperfusion alone is not sufficient to rescue dying cells due to the activation of injury-related pathways. Thus, there is an unmet need for the identification of post-ischemic injury mechanisms to develop effective stroke treatment. Axonal injury is an important independent risk factor and burden for adverse outcomes following a stroke, even in intravenous thrombolysis patients (Curtze et al., 2015). It is crucial, therefore, to search for therapeutic options that protect the entire brain by treating both gray and WM components against ischemia.

### Mechanisms of Ischemic White Matter Injury Are Age-Dependent

Mechanisms underlying ischemic WM injury prove to be unexpectedly complex and distinct from gray matter (GM) injury (Wrathall et al., 1994; Agrawal and Fehlings, 1997; Fern and Ransom, 1997; McDonald et al., 1998; Sanchez-Gomez and Matute, 1999; Follett et al., 2000; Tekkök and Goldberg, 2001; Stys, 2004; Tekkök et al., 2007). WM injury mechanisms follow a spatiotemporal sequence of events; axons are injured directly by the loss of ionic homeostasis resulting in toxic accumulation of intracellular Na^+^ and Ca^2+^ (Stys et al., 1990; Fern et al., 1995; Wolf et al., 2001; Ouardouz et al., 2003; Underhill and Goldberg, 2007), while astrocytes due to reversal of Na^+^-dependent glutamate transporters release excessive glutamate (Tekkök et al., 2007) leading to injury of oligodendrocytes and the myelin they produce (Matute et al., 1997; McDonald et al., 1998; Li et al., 1999; Sanchez-Gomez and Matute, 1999; Tekkök and Goldberg, 2001; Alberdi et al., 2002; Micu et al., 2006; Tekkök et al., 2007). Consistent with this idea, removal of extracellular Ca^2+^, blockade of AMPA/kainate receptors, or blockade of reverse glutamate transport reduces ischemic WM injury. Moreover, glutamate accumulation triggers oxidative injury pathways by competing with cysteine. Together with mitochondrial dysfunction and nitric oxide synthetase (NOS) activation, reactive oxidative stress (ROS) production increases contributing to irreversible ischemic WM injury.

Aging is the most independent risk factor for stroke. Age-related changes in the molecular structure of WM dictate injury mechanisms by surpassing the ionic pathway and initiating injury by combined excitotoxic and oxidative injury pathways. Consequently, protective interventions in young WM become ineffective at promoting recovery of, or even injurious to, aging WM (Saab et al., 2016; Bastian et al., 2019a). For instance, in the aging axons, there is a significant increase in glutamate transporter-1 (GLT-1) levels leading to excessive extracellular glutamate accumulation presumably due to an increased need for glutamate signaling in aging WM to maintain its function (Baltan, 2009; Baltan et al., 2011). However, these adaptive changes act against the tissue by causing glutamate toxicity and mitochondrial energy depletion in aging axons during an ischemic episode (Stahon et al., 2016). To maintain proper axon function, axonal mitochondria exhibit unique and complex dynamics to proficiently buffer Ca^2+^, produce sufficient ATP, and effectively scavenge ROS. Ca^2+^ overload activates eNOS to produce nitric oxide (NO) and ROS, which are proposed as diffusible second messengers to link oligodendrocyte excitotoxicity to axon injury (Matute, 2010; Voccoli et al., 2014; Bastian et al., 2018). The fusion and fission processes of mitochondria are delicately regulated to coordinate the spatiotemporal properties of mitochondrial Ca^2+^ responses and the physiological and pathophysiological consequences of Ca^2+^ signals (Baltan, 2014b). By enhancing fusion or inhibiting fission, elongated mitochondria efficiently buffer Ca^2+^, thus preventing eNOS activation and subsequent ROS production (Lugus et al., 2011; Miller et al., 2013; Baltan, 2014b; Bastian et al., 2018). In aging axons, there is an increase in mitochondrial fusion, presumably to effectively buffer increased Ca^2+^ load and ROS production, which further alters the mitochondrial dynamics and function (Baltan, 2009; Stahon et al., 2016). Therefore, an age-dependent modification in mitochondrial bioenergetics may underlie the increased vulnerability of aging axons to ischemia. The intimate link between changes in aging WM structure and response to injury complicates the development of possible therapeutic options and warrants attention to identify beneficial interventions that act on shared molecular targets between young and aging WM.

### Casein Kinase 2 Mediates Ischemic White Matter Injury

The optic nerve, a purely myelinated CNS WM tract, offers several advantages to study the mechanisms of WM injury. These advantages include minimal surgical injury due to isolation techniques, preservation of the three-dimensional structure of myelinated axons with their supporting glia, stable and quantifiable recording of action potentials for prolonged periods (Cavallotti et al., 2002; Cavallotti et al., 2003). Tissue collected at the end of the experiments can be further processed to quantify the proteins of interest. In addition, in fixed optic nerve tissue, the cellular and axonal structures can be immunolabeled with cell-specific antibodies or prepared for three-dimensional electron microscopy imaging for ultrastructural assessment. The corpus callosum (CC) is another WM tract and offers important advantages for the investigation of *in vitro* CC slices to quantify axon function and *in vivo* WM (selective WM ischemic by stereotaxic L-NIO injections) injury which can be assessed with behavioral tests (Nunez et al., 2016). These two WM tracts allow an excellent combined function-structure analysis of glial cells and axons after stroke.

**FIGURE 1:**
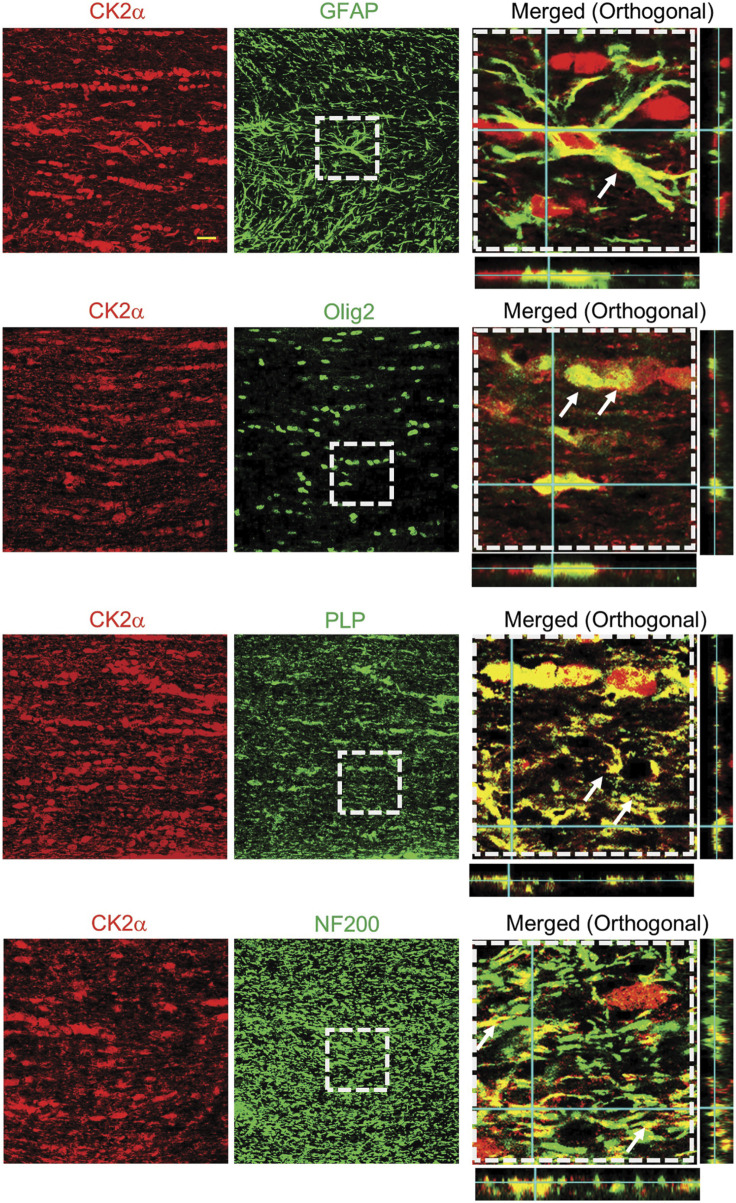
CK2α expression and localization in mouse optic nerves. CK2α subunits are expressed in optic nerve astrocytes, myelin sheath, and oligodendrocytes. To identify the cellular expression of CK2α subunits in the mouse optic nerve, CK2α was co-immunolabeled with glial fibrillary acidic protein (GFAP, astrocytes, top row), oligodendrocyte lineage transcription factor 2 (Olig2, oligodendrocytes, second row), myelin proteolipid protein (PLP, myelin, third row), and neurofilament protein (NF200, axons, bottom row). Note that the merged images (xy, xz and yz orthogonal view) in the right panels are enlarged areas (50 μm × 50 μm) indicated by the squares with dashed lines in the middle panels. Scale bar = 20 μm. (Figure from Bastian et al., 2019).

**FIGURE 2:**
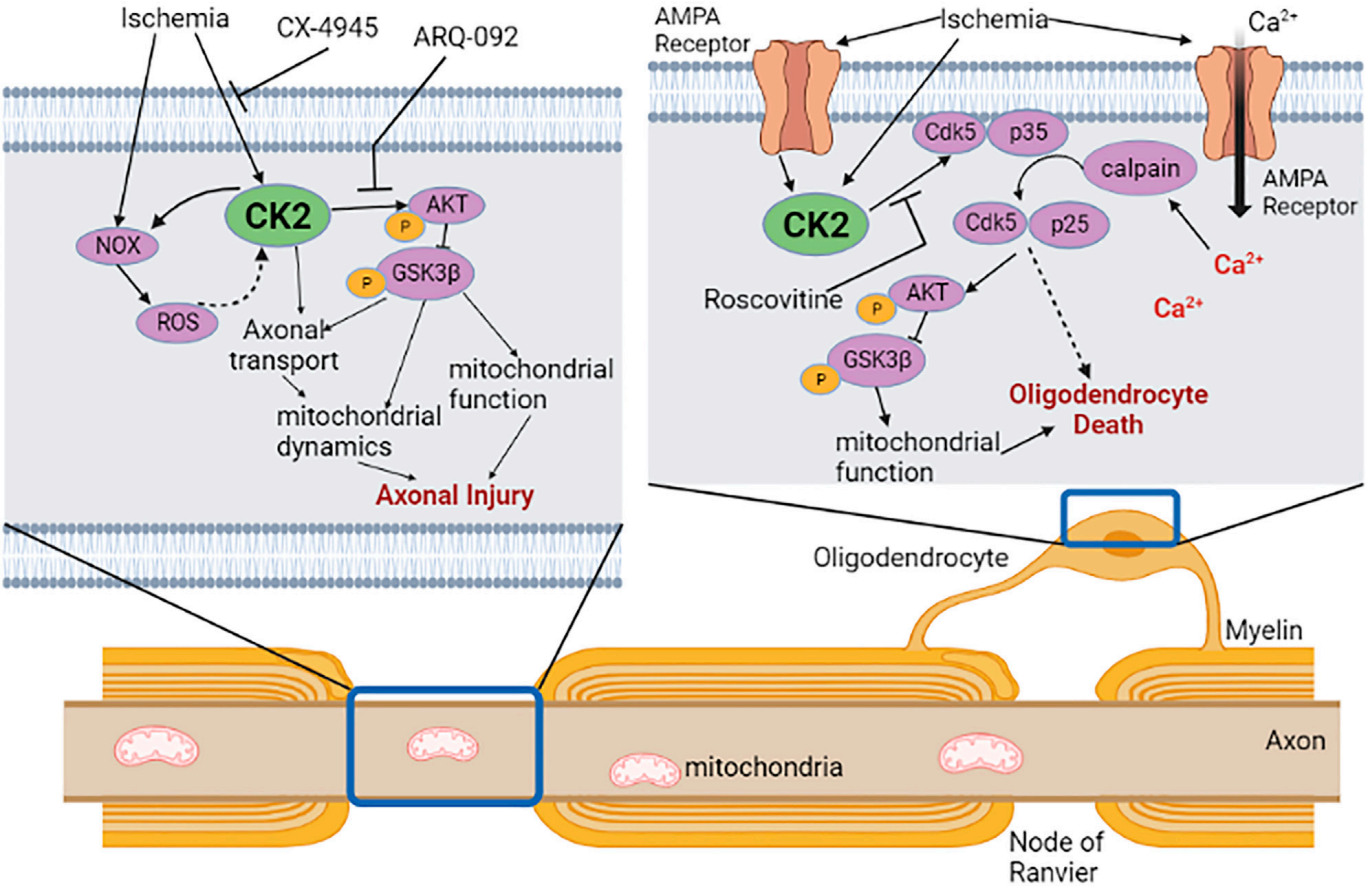
CK2 signaling in white matter ischemia. In axon, ischemia can directly activate CK2 or NADPH oxidase (NOX) to increase reactive oxygen species (ROS). CK2 then activates Akt/GSK3β signaling through phosphorylation to disrupt axonal mitochondrial dynamics and function. Specific CK2 inhibitor CX-4945 or phosphorylated Akt inhibitor ARQ-092 confers axonal protection when applied during or after ischemia. In oligodendrocytes, ischemia activates CK2 directly or via activation of AMPA receptors leading to an influx of Ca^2+^. Ca^2+^ activates calpain, which subsequently acts on Cdk5/p35 complex to untether Cdk5/p25 complex from the membrane that can then phosphorylate Akt/GSK3β in oligodendrocytes. Roscovitine, an inhibitor of Cdk5, improves axon function recovery following ischemia, presumably through protection of oligodendrocytes and/or axons. The effect of untethered Cdk5 on oligodendrocyte injury is yet to be investigated. Dotted arrow indicates potential interactions. Created with Biorender.com.

We evaluated the expression and localization of CK2α in mouse optic nerves (MONs), using isoform-specific antibodies to support a biological basis for investigating CK2 signaling in WM (Figure 1). CK2α is expressed in axons and glial cells in MONs demonstrated by colocalization of CK2α subunit with GFAP (+) astrocyte nuclei and some processes, NF-200 (+) axons, Olig2 (+) oligodendrocytes, and PLP (+) myelin. The robust expression pattern of CK2α suggests an extensive kinase regulation of WM structure and function (Moreno et al., 1999; Brechet et al., 2008; Yoshimura and Rasband, 2014; Rosenberger et al., 2016). Indeed, CK2 signaling is different under physiological and ischemic stress conditions. Under normal physiological conditions, CK2 signaling is important for the clustering of Na^+^ channels at axon initial segments and nodes of Ranvier to enable and preserve axonal excitability (Brechet et al., 2008; Hien et al., 2014) and for oligodendrocyte development (Huillard et al., 2010). On the other hand, during ischemia, CK2 signaling mediates injury to glial cells and impairs axon function either directly and/or through Cdk5 and PTEN/AKT/GSK3β signaling regulation of downstream effectors (Figure 2). Subsequently, CK2 blockade preserves oligodendrocytes and axonal mitochondrial integrity, dynamics, and function (Baltan et al., 2018; Bastian et al., 2019b). Changes in signaling due to ischemia are achieved by either increasing CK2 activity (Charriaut-Marlangue et al., 1991; Hu and Wieloch, 1993; Hase et al., 2005; Chao et al., 2007) or by the movement of CK2 to a different subcellular compartment (Serrano et al., 1989; Charriaut-Marlangue et al., 1991; Diaz-Nido and Avila, 1992; Faust et al., 2001; Qaiser et al., 2014). CK2 can directly interfere with mitochondrial axonal transport (Pigino et al., 2009) to ultimately alter mitochondrial dynamics (Amiri and Hollenbeck, 2008; Liu et al., 2009; Twig et al., 2010) and function (Detmer and Chan, 2007). Another signaling pathway that CK2 regulates is Cdk5 (Lim et al., 2004) (Figure 2). Both CK2 and Cdk5 are expressed at nodes of Ranvier (Brechet et al., 2008; Cerda and Trimmer, 2011) and by oligodendrocytes (Huillard et al., 2010; Yang et al., 2013), where CK2 can inhibit Cdk5 by contact inhibition (Lim et al., 2004). Therefore, for Cdk5 to be activated, CK2 must move away from Cdk5. Cdk5 is tethered to the membrane by its association with p35, a protein with a membrane-anchoring domain. The Cdk5/p35 complex is then fully activated by Ca^2+^-dependent proteolytic cleavage of p35 to p25 by calpain, effectively removing its membrane-anchoring domain (Meyer et al., 2014). Local intracellular Ca^2+^ may be increased by the reversal of the Na^+^-Ca^2+^ exchanger or by activation of L-type Ca^2+^ channels (Brown, 2001; Baltan, 2009). The Cdk5 complex can then move from nodes of Ranvier to other cellular compartments to control AKT signaling (Lou et al., 2008) and axonal transport (Shea et al., 2004) to ultimately regulate mitochondrial dynamics (Amiri and Hollenbeck, 2008; Liu et al., 2009; Twig et al., 2010) and function (Detmer and Chan, 2007) (Figure 2). In addition, CK2 can regulate PTEN/AKT/GSK3β (Silva et al., 2008; Silva et al., 2010) signaling. CK2 inhibits PTEN by phosphorylation, which leads to the activation of AKT. AKT phosphorylates GSK3β to inhibit its activity (Cross et al., 1995), which could lead to changes in mitochondrial transport (Embi et al., 1980; Cohen and Frame, 2001), glycogen synthase (Embi et al., 1980; Cohen and Frame, 2001), and mitochondrial function (Detmer and Chan, 2007; Amiri and Hollenbeck, 2008; Liu et al., 2009; Twig et al., 2010).

Our recent studies, for the first time, establish that a PK mediates ischemic injury in WM by CK2 signaling leading to activation of Cdk5 and PTEN/AKT/GSK3β pathways (Bastian et al., 2019b) (Figure 2). To identify the role of CK2 signaling in WM ischemic injury, we followed the preclinical recommendations of the Stroke Therapy Academic Industry Roundtable (STAIR) (Fisher et al., 2009). Based on this set of criteria, we use CX-4945 (Silmitasertib), a highly *selective* (Siddiqui-Jain et al., 2010; Son et al., 2013)*, specific* (Battistutta et al., 2011)*,* and *potent* CK2 inhibitor that can be administered *orally* (Ryan et al., 2005; Zhong et al., 2020) and *crosses the blood-brain barrier* (Vita et al., 2005; Sallam et al., 2008; Cheng et al., 2012; Zheng et al., 2013). CX-4945 is an *FDA-approved* anti-cancer drug (Martins et al., 2014). CX-4945 exerts dose-dependent protection to axon function (max protection at 5 µM) and the lack of any baseline effects of this drug on axon conduction allows comparison of recovery in *in vitro* experiments and behavioral assessments in *in vivo* experiments. Thus, we propose that CK2 inhibition protects the brain against ischemia by protecting axonal and glial compartments.

Equally important, CX-4945 confers similar protection to aging (12–14 months) and old (>20 months) WM when applied after ischemia promoting axon function recovery. The effects of aging on myelinated axons are more complicated and extensive than those in cortical GM. Despite larger and thicker aging axons, with longer and thicker mitochondria that correlate with lower ATP production, CX-4945 still provides post-ischemic protection to aging axon function by preserving mitochondrial integrity. We, therefore, suggest that CK2 signaling is a shared pathway underlying WM injury independent of age.

An important outcome measure emphasized by STAIR criteria is the consideration of the female sex. Stroke in females is associated with a decreased likelihood of excellent outcome after acute ischemic stroke, particularly in older age groups. There is a correlation between markers of WM integrity and functional outcomes in women, which implies a potential sex-specific WM injury mechanism (Etherton et al., 2017; Phan et al., 2018; Etherton et al., 2019). Therefore, evaluation of CK2 signaling in female WM injury and whether CK2 inhibition provides equal protection compared to male WM is a pending goal of our group.

### Casein Kinase 2 Mediates Post-Ischemic WM Injury By Selectively Acting on the AKT Pathway

The finding that CK2 inhibition with CX-4945 when applied before or after the end of an ischemic episode promotes WM functional recovery raises the question of whether Cdk5 or AKT activation plays a distinct role in conferring post-ischemic WM protection (Figure 2). Inhibition of Cdk5 using Roscovitine protects axon function only when applied during ischemia, mainly acting on oligodendrocytes and axons (Figure 2). On the other hand, inhibition of the active conformation of AKT is beneficial when applied during or after ischemia suggesting that a window of opportunity exists in ameliorating ischemic injury in WM. AKT is involved in many neurological processes, and AKT isoforms are distinct regarding their tissue expression, pathway activation, and inhibitor sensitivity (Shaw and Kirshenbaum, 2006; Skeen et al., 2006). However, very few studies have examined AKT isoform expression at the cellular level, and cell- and age-specific AKT isoforms expression in WM remains unknown. Therefore, a systematic investigation of AKT isoforms and their contribution to axon and glia function is warranted.

### Casein Kinase 2 Disrupts Axonal Mitochondria

We recently showed that the preservation of mitochondrial integrity is an essential component of post-ischemic protection of axon function in WM. (Baltan et al., 2011; Baltan, 2014a; Stahon et al., 2016). CX-4945 promotes young and aging axon function recovery following ischemia by preserving axonal mitochondria. Cdk5 directly impacts mitochondrial dynamics and function by increasing the production of ROS and phosphorylation of the mitochondrial fission protein Drp-1, leading to mitochondrial dysfunction (Sun et al., 2008; Morel et al., 2010; Cherubini et al., 2015; Jahani-Asl et al., 2015; Klinman and Holzbaur, 2015; Park et al., 2015). However, Cdk5 inhibition fails to exert post-ischemic protection to axon function, implying that Cdk5 signaling is important to alleviate oxidative injury specifically during ischemia. The finding that selective inhibition of phosphorylated AKT confers post-ischemic protection to axon function proposes a novel role for PTEN/AKT signaling in mediating mitochondrial disruption after an ischemic episode in WM. AKT activation contributes to increased glutamate release during OGD and ATP depletion, as well as enhanced excitotoxicity (Baltan et al., 2008) due to the upregulation of GLT-1 expression in astrocytes (Li et al., 2006; Ji et al., 2013; Zhang et al., 2013). As a result, the application of ARQ-092, which is a specific blocker for the active form of AKT (Yu et al., 2015; Lapierre et al., 2016), promotes axon function recovery suggesting that the active conformation of AKT is an important molecular target for post-ischemic protection of axon function (Figure 2). Moreover, the GSK3β isoform which is a part of the AKT/GSK3β signaling cascade has been reported to be a significant therapeutic target for cerebral ischemia (Koh S.-H. et al., 2008; Cowper-Smith et al., 2008). GSK3β inhibition decreases mitochondrial ROS production and prevents neuronal damage establishing an interesting relationship between GSK3β and mitochondria (Valerio et al., 2011). Because, we also observed that CK2 inhibition improved axon function recovery by decreasing the inactivation of GSK3β in WM, these findings suggest that GSK3β could be a common target to protect both GM and WM after ischemic stroke.

### Casein Kinase 2 Mediates WM Injury By Regulating Micro RNAs (miRNAs)

The miRNAs emerge as important mediators of neuronal injury during an ischemia attack. However, the role and involvement of miRNAs remain unestablished in WM ischemic injury. Therefore, in our recent study, we characterized miRNA profiles in optic nerve following ischemia using the NanoString nCounter^®^ miRNA Expression Panel. Together with *in situ* hybridization, and *in silico* KEGG pathway analysis, we show that the most abundant miRNAs in the optic nerve are expressed in astrocytes, and oxygen-glucose deprivation (OGD) differentially regulates miRNA expression in the optic nerve (Baltan et al., 2021).

Based on our analysis, it remains challenging to define whether miRNAs regulated by ischemia in WM are beneficial or detrimental to the recovery of axon function. Some miRNAs were reciprocally regulated by OGD or OGD and CX-4945 application. For instance, OGD and CX-4945 selectively modulated miR-1937a, miR-1937b, miR-1959, miR-200a, miR-501-3p, and miR-654-3p (Table 1) (Baltan et al., 2021). We propose that these miRNAs may be associated with the beneficial effects of CX-4945. In agreement, miR-501-3p is expressed in neurons which in optic nerve (pure WM tract without neuronal cell body) infers axonal expression (Baltan et al., 2021). Because CK2 inhibition promotes axon function by preserving axonal mitochondria (Bastian et al., 2019b), we hypothesize that CK2 inhibition exerts white matter protection by regulating miR-501-3p. Particularly, because miR-501-3p is shown to mediate the regulation of GluA1 subunit expression of AMPA receptors and the subsequent mitochondrial injury (Sripada et al., 2012). Furthermore, miR-501-3p is expressed in human and reported to be a novel serum biomarker, which relates to the severity of Alzheimer’s Disease (Hara et al., 2017). Hence, miR-501-3p appears as a promising target for further investigation. In addition, we determined that exosomal expression of miR-1959 and miR-1937b in astrocytes is also differentially regulated by OGD or OGD and CX-4945 (Jovičić and Gitler, 2017). Astrocytic exosomes are 50—100 ŋm membrane-bound vesicles, which contain and transfer a selected group of miRNAs to other cells. This suggests that astrocytes coordinate an efficient communication among glia cells in response to OGD or CK2 inhibition during ischemia in WM. It will be intriguing to identify the role and cellular target of miR-1959 and miR-1937b in WM. Another novel finding is that CX-4945 treatment affects some common KEGG signaling pathways. One of these signaling pathways is the ErbB signaling system (Li et al., 2020) which is important to maintain axon function as well as supporting glia and myelin. Expectedly, a disruption in ErbB signaling may impair axon function by causing myelin damage (Carroll et al., 1997). Additionally, ErbB signaling in neurons and macrophages/microglia determines neuroprotection and repair capacity after ischemia (Xu and Ford, 2005). Interestingly, CX-4945 regulates Wingless/int1 (Wnt) signaling, which is involved in neurogenesis after cerebral ischemia, implicating Wnt signaling as a therapeutic target for ischemic injury (Yu et al., 2018; Qiu et al., 2019). CX-4945 also regulates mTOR and axon guidance pathways that are important for neuroprotection after an ischemic stroke (Sofer et al., 2005; Koh P.-O. et al., 2008; Foster and Fingar, 2010; Koh, 2010; Hinman, 2014). However, further experiments are needed to validate whether the mechanisms of protection of CX-4945 are indeed mediated through these signaling pathways.

**TABLE 1:** List of miRNAs with fold changes after OGD and OGD with CK2 inhibition and their cellular expression.

OGD			
MiRNA	Fold Change	*p*-value	Cellular Expression
A			
miR-1959	1.70	0.032	Astrocyte exosomes
miR-501-3p	1.58	0.025	Neurons
miR-146b	1.57	0.049	Neurons, Astrocytes, Oligodendrocytes
miR-201	1.50	0.027	NA
miR-335-3p	1.50	0.031	Neurons, Astrocytes
miR-1937a	−1.50	0.035	NA
miR-1937b	−1.50	0.035	Astrocyte exosomes
B			
OGD+CX-4945			
MiRNA	Fold Change	*p*-value	Cellular Expression
miR-1937a	1.53	0.030	NA
miR-1937b	1.53	0.030	Astrocyte exosomes
miR-m01-2	−1.63	0.002	NA
miR-501-3p	−1.65	0.047	Neurons
miR-200a	−1.69	0_._047	Neurons, Astrocytes, Oligodendrocytes
miR-1959	−1 R4	0 025	Astrocyte exosomes
miR-654-3p	−2.24	0.011	NA

(A) OGD, compared to control. (B) OGD + CK2 inhibition with CX-4945, compared to OGD., Welch’s *t*-test. Negative sign indicates decrease. [Modified from (Baltan et al., 2021)].

## Discussion

WM ischemic lesions are correlated to neurological deficits (Yamada et al., 2003; Sea Lee et al., 2005) and particularly the extent and localization of WM injury may dictate functional deficits and recovery in humans. Because the rodent brain has a relatively small WM (10–15%) (Zhang and Sejnowski, 2000) and most widely used stroke models spare corpus callosum, the injury mechanisms mostly provide information about neuronal populations. Neuroprotective approaches focused solely on neuronal survival may be one of the reasons for the failure in translating experimental findings successfully to clinical applications. It is crucial to consider WM integrity in experimental models to identify ideal therapeutic targets for stroke patients.

In summary, our recent studies provide evidence that CK2 signaling activates Cdk5 and AKT/GSK3ß signaling pathways to mediate WM ischemic injury. The downstream molecular pathways are activated in a spatiotemporal way such that Cdk5 signaling becomes significant during ischemia, while AKT signaling emerges as the key pathway during the post-ischemic period. Consistent with this, inhibition of CK2 or the activated form of AKT confers post-ischemic protection to axon function and promotes recovery in young and aging WM. The protective effects of CK2 inhibition correlate with the conservation of oligodendrocytes, axon structure, and axonal mitochondria. Several miRNAs are differentially regulated by CX-4945 compared to ischemia, and these miRNAs may participate in ischemic WM injury mechanisms. MiRNAs are promising candidates for biomarkers of injury and therapeutic interventions as they are readily detected in body fluids. We also show that CX-4945 regulates a group of murine-associated viral miRNAs (for example see Table 1) which may justify the use of CX-4945 in clinical trials for Covid19 patients (Baltan et al., 2021) (ClinicalTrials.gov Identifier: NCT04663737). Finally, our findings may have mechanistic and therapeutic implications for dementia, Alzheimer’s disease, multiple sclerosis, periventricular leukomalacia, and Parkinson’s disease that involve WM injury.
